# Metabolome response to temperature-induced virulence gene expression in two genotypes of pathogenic *Vibrio parahaemolyticus*

**DOI:** 10.1186/s12866-016-0688-5

**Published:** 2016-04-26

**Authors:** Bo Feng, Zhuoran Guo, Weijia Zhang, Yingjie Pan, Yong Zhao

**Affiliations:** College of Food Science and Technology, Shanghai Ocean University, No. 999 Hu Cheng Huan Road, Shanghai, China; Shanghai Engineering Research Center of Aquatic-Product Processing & Preservation, Shanghai, China; Laboratory of Quality & Safety Risk Assessment for Aquatic Products on Storage and Preservation (Shanghai), Ministry of Agriculture, Shanghai, China

**Keywords:** *Vibrio parahaemolyticus*, Relative virulence gene expression, Metabolic profiling, Pearson’s correlation analysis

## Abstract

**Background:**

*Vibrio parahaemolyticus* is a main causative agent of serious human seafood-borne gastroenteritis disease. Many researchers have investigated its pathogenesis by observing the alteration of its virulence factors in different conditions. It was previously known that culture conditions will influence the gene expression and the metabolic profile of *V. parahaemolyticus*, but little attention has been paid on the relationship between them. In this study, for the first time, the metabolomics response in relation to the expression of two major virulence genes, *tdh* and *trh*, induced at three temperatures (4, 25 and 37 °C) was examined in two genotypes of pathogenic *Vibrio parahaemolyticus* (ATCC33846 (*tdh+/trh−/tlh+*) and ATCC17802 (*tdh−/trh+/tlh+*)).

**Results:**

Reverse transcription real-time PCR (RT-qPCR) analysis illustrated that the expression levels of *tdh* and *trh* induced at 25 °C in *V. parahaemolyticus* were significantly higher than those induced at 4 and 37 °C. Principal components analysis (PCA) based on the UPLC & Q-TOF MS data presented clearly distinct groups among the samples treated by different temperatures. Metabolic profiling demonstrated that 179 of 1,033 kinds of identified metabolites in ATCC33846 changed significantly (*p* <0.01) upon culturing at different temperatures, meanwhile 101 of 930 kinds of metabolites changed (*p* <0.01) in ATCC17802. Pearson’s correlation analysis highlighted the correlation between metabolites and virulence gene expression levels. At the threshold of | r | = 1, *p* <0.01, 12 kinds of metabolites showed extremely significant correlations with *tdh* expression, and 4 kinds of metabolites significantly correlated with *trh* expression. It is interesting that 3D, 7D, 11D-Phytanic acid showed the same trend with pyrophosphate, whose derivative could activate the degradation of phytanic acid. Several metabolites could be sorted into the same class by the method of chemical taxonomy, by assuming that they are involved in the same metabolic pathways.

**Conclusions:**

This research can help to find biomarkers to monitor virulence gene expression*,* and can further help laboratory and clinical research of *V. parahaemolyticus* from the perspective of metabolomics.

**Electronic supplementary material:**

The online version of this article (doi:10.1186/s12866-016-0688-5) contains supplementary material, which is available to authorized users.

## Background

*Vibrio parahaemolyticus* is a gram-negative and halophilic bacterium, known as a leading cause of seafood-borne poisoning all over the world [1–3]. Numerous outbreaks of food-borne disease were associated with *V. parahaemolyticus* infection [4–6]. Most people are infected by eating raw or undercooked shellfish, particularly oysters. *V. parahaemolyticus* can also cause systemic infection through wound infection [7].

The pathogenesis of *V. parahaemolyticus* is complex. As we known, *V. parahaemolyticus* strains contain a number of different virulence factors including adhesins, thermostable direct hemolysin (TDH), TDH-related hemoysin (TRH), two type III secretion systems, T3SS1 and T3SS2 [8, 9]. Two co-existed type VI secretion systems, T6SS1 and T6SS2, would be new virulence factors of *V. parahaemolyticus* [10, 11]*.* Previous studies have found that pathogenic *V. parahaemolyticus* often carries thermostable direct hemolysin (*tdh*) and/or thermostable-related hemolysin (*trh*) genes [12–14]. TDH and TRH were first identified as *V. parahaemolyticus* virulence factors in the 1980s, and then considered as the main toxins which induce cytotoxicity and enterotoxicity [15–17]. In recently years, though research about regulatory mechanism of virulence has been discovered [18], there were few discussions about the correlation between growth condition and virulence gene expression of *V. parahaemolyticus*. Studies of virulence factors have made remarkable progress, while the synergy effect and pathogenicity of them are still under investigation [9].

The rapid development of metabolomics introduced a powerful way to study the pathogenesis of diseases by analyzing the metabolites of patients and monitoring the alteration of biomarkers in the course of diseases [19]. Metabolomics could be valued as a new vision for characterization of a pathogen during its growth and infection process. It has been confirmed that there is correlation between genes and metabolites in *E. coli* [20], but little attention has been paid to the analysis of the metabolome for better understanding the pathogenesis of *V. parahaemolyticus*. So it is meaningful to research the virulence gene expression, the metabolic situation of *V. parahaemolyticus*, and most significantly, the correlationship between them.

Previous research of our group has detected *tdh* and *tlh* expression in *V. parahaemolyticus* by reverse transcription real-time PCR (RT-qPCR) [21] and distinguished different pathogenic *V. parahaemolyticus* strains based on metabolic profiling [22]. This research focused on investigating the correlation between the virulence gene expression of *V. parahaemolyticus* and its metabolites induced at three temperatures, for better understanding its pathogenesis and monitoring typical virulent *V. parahaemolyticus* strains. Three temperatures, 4, 25 and 37 °C, were chosen as the incubation temperatures for simulating the storage temperature of fresh aquatic products, environmental temperature and human body temperature. The *tdh* and *trh* gene were chosen as the virulence genes to be studied in this research. Relative virulence gene expression of two standard pathogenic strains of *V. parahaemolyticus*, ATCC33846 (*tdh*+/*trh*−/*tlh*+) and ATCC17802 (*tdh*−/*trh*+/*tlh*+), were performed by RT-qPCR. Metabolic profiling of *V. parahaemolyticus* was determined by Ultra Performance Liquid Chromatography & Quadrupole-Time-of-Flight Mass Spectrometry (UPLC & Q-TOF MS). Furthermore, the relationship between metabolome and virulence gene expression was explored by Pearson’s correlation analysis.

## Results

### Virulence gene expression of *V. parahaemolyticus*

**Fig. 1:**
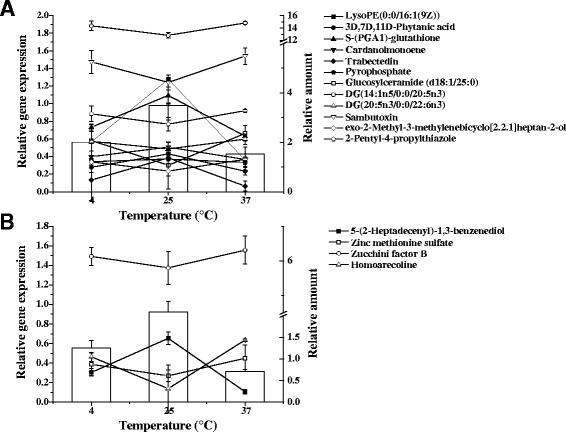
Relative quantifications of gene expressions and metabolites in *V. parahaemolyticus*. **a** *tdh* gene expression (column) and 12 kinds of metabolites (line) which had extremely high correlation with *tdh* gene expression (| r | = 1, *p* <0.01) in ATCC33846. Relative amount of six kinds of metabolites illustrated positive correlation with relative gene expression under different temperatures; the other six kinds of metabolites showed negative correlation. **b** *trh* gene expression (column) and four kinds of metabolites (line) which had extremely high correlation with *trh* gene expression (| r | = 1, *p* <0.01) in ATCC17802. One metabolite illustrated positive correlation with gene expression; the other three metabolites showed negative correlation

The virulence gene expression of two genotypes of *V. parahaemolyticus* were investigated by RT-qPCR under different culture conditions (4, 25 and 37 °C). Both *pvuA* and *pvsA* were used as the reference genes due to the bias which may be caused by the fluctuation in expression level of a single reference gene [23]. The expression levels of *tdh* induced at 25 °C in *V. parahaemolyticus* ATCC33846 (*tdh*+/*trh*−/*tlh*+) were approximately two-fold higher than those induced at 4 and 37 °C (*p* <0.05) (Fig. 1a). The expression of the *trh* gene presented the same trend in *V. parahaemolyticus* ATCC17802 (*tdh*−/*trh*+/*tlh*+) (Fig. 1b).

### Metabolic profiling of *V. parahaemolyticus*

**Fig. 2:**
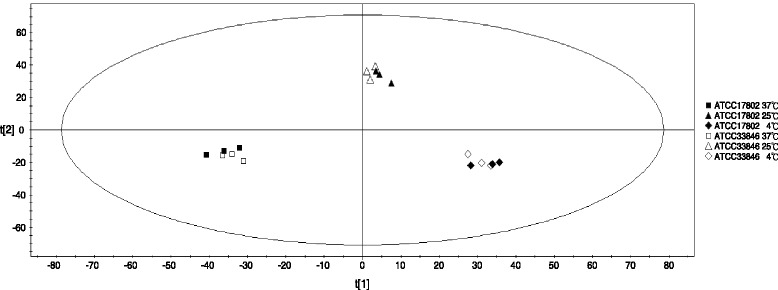
Principal component analysis score plot. First and second PCs from metabolites found in *V. parahaemolyticus* grown at 4, 25 and 37 °C. (Based on measurements of three independent biological replicates)

To generate an overview of the data set, the positive and negative ions were detected and processed by MassLynx 4.1, and then the data were fed to SIMCA-P 11.5 for principal components analysis (PCA). PCA was performed to validate the differences between the metabolites in *V. parahaemolyticus* affected by different temperatures. Figure 2 showed the scatter plot using the score of first principal component (PC1) and the second principal component (PC2) for each sample. In general, the *V. parahaemolyticus* samples were clustered in three distinct groups according to temperatures (4, 25 and 37 °C). The close clustering of the *V. parahaemolyticus* samples indicated their high similarity in terms of their metabolites compositions and abundances.

**Fig. 3:**
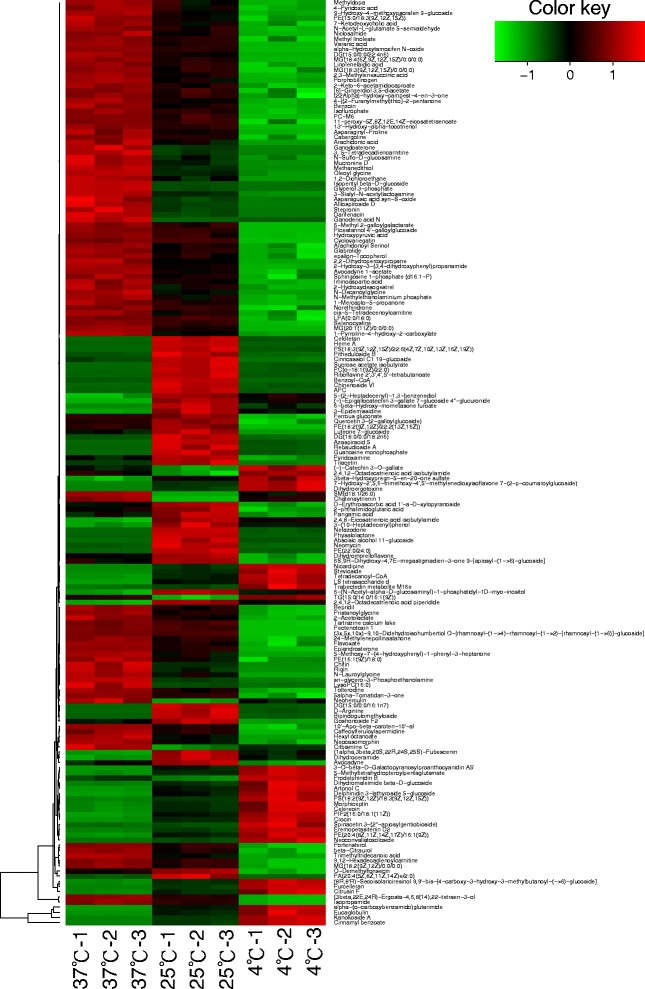
Heatmap of the metabolite whose concentration changed significantly (*p* <0.01), grouped by different culturing temperatures in ATCC33846. Colors represent an increase and decrease of metabolite (see color key). The dendrogram for metabolite clustering is shown on the left

**Fig. 4:**
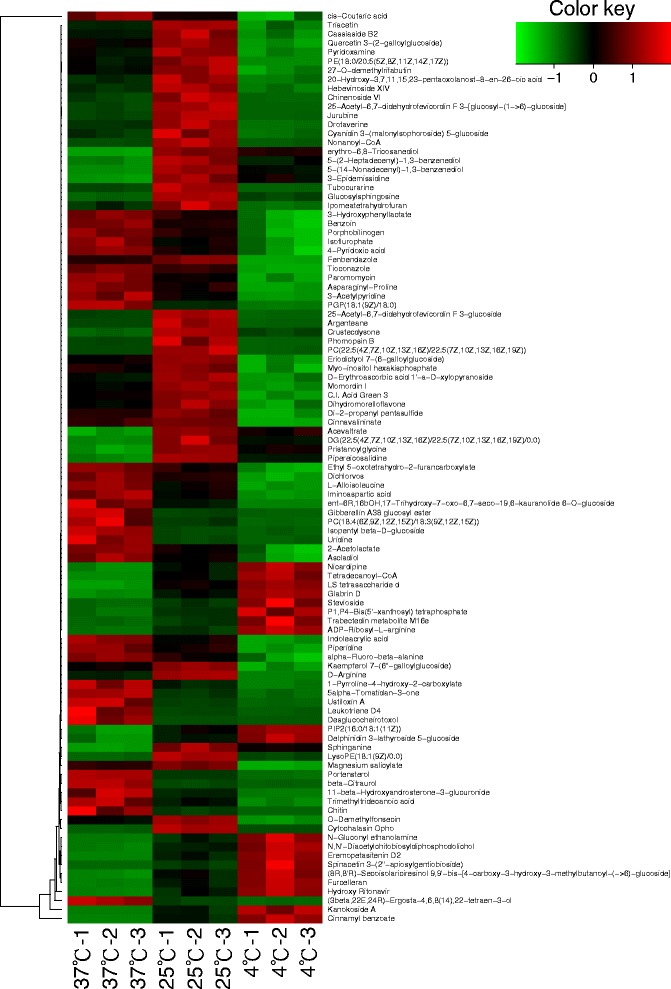
Heatmap of the metabolite whose concentration changed significantly (*p* <0.01), grouped by different culturing temperatures in ATCC17802. Colors represent an increase and decrease of metabolite (see color key). The dendrogram for metabolite clustering is shown on the left

Typical UPLC & Q-TOF MS chromatograms of *V. parahaemolyticus* were analyzed. According to UPLC-MS data, over 5000 peaks were determined in the two strains of bacteria grown at 4, 25 and 37 °C. Every peak was identified as a certain metabolite by searching its value of m/z in HMDB. The value of relative concentration of metabolite was used for statistical analysis (Additional file 1: Table S1 and Additional file 2: Table S2). The number of identified peaks, total numbers of identified metabolites, and numbers of metabolites changed significantly and numbers of metabolites highly correlated with *tdh/trh* expression level were shown in Table 1. There are 179 and 101 kinds of metabolites changed with incubation temperature significantly in ATCC33846 and ATCC17802, respectively. Heatmaps were employed to visualize the variations of metabolites concentration, which containing hierarchical clustering on the left (Figs. 3 and 4). Metabolites which were arranged nearby have similar variational rules of concentrations in different temperatures. The significantly changed metabolites could be classified into different categories by chemical taxonomy, such as alkaloids and derivatives, benzenoids, lipids and lipid-like molecules, nucleosides, nucleotides, and analogues, organic acids and derivatives, organoheterocyclic compounds, phenylpropanoids and polyketides (Additional file 3: Table S3 and Additional file 4: Table S4).

**Table 1:** Metabolic profiling of *V. parahaemolyticus* grown at different temperatures

Strains	Numbers of peaks identified at different temperatures			Total numbers of metabolites identified	Numbers of metabolites changed significantly at different temperatures (*p* <0.01)	Numbers of metabolites highly correlated with *tdh/trh* expression level (|r| >0.8)
	4 °C	25 °C	37 °C			
ATCC33846	882	1,011	916	1,033	179	388
ATCC17802	847	887	868	930	101	345

### Correlation between expression of *tdh*/*trh* and metabolome

In previous studies, Pearson’s correlation analysis was applied in examining the correlation between mRNA and protein abundance [24, 25]. In this study, *tdh* and *trh* gene expression were used for discussing the correlation with metabolites of ATCC33846 and ATCC17802, respectively. There were 388 (37.6 %) and 345 (37.1 %) kinds of metabolites that showed high correlationship (| r | >0.8) with the gene expression of *tdh* and *trh* in ATCC33846 and ATCC17802, respectively (Table 1).

Following, the threshold of | r | = 1, *p* <0.01 was chosen for further insight into the relationship between the virulence gene expression and metabolome. There were 12 and four kinds of metabolites that showed extremely significant correlation with *tdh* and *trh* gene expression level in ATCC33846 and ATCC17802, respectively (Table 2).

**Table 2:** The metabolites highly correlated with the expression level of *tdh* and *trh* genes

Gene	Compounds	Correlation coefficient	*p*-value
*tdh*	LysoPE(0:0/16:1(9Z))	1.000^a^	0.001
*tdh*	3D,7D,11D-Phytanic acid	1.000^a^	0.002
*tdh*	S-(PGA1)-glutathione	1.000^a^	0.003
*tdh*	Cardanolmonoene	1.000^a^	0.004
*tdh*	Trabectedin	1.000^a^	0.006
*tdh*	Pyrophosphate	1.000^a^	0.008
*tdh*	Glucosylceramide (d18:1/25:0)	−1.000^a^	0.001
*tdh*	DG(14:1n5/0:0/20:5n3)	−1.000^a^	0.004
*tdh*	DG(20:5n3/0:0/22:6n3)	−1.000^a^	0.005
*tdh*	Sambutoxin	−1.000^a^	0.006
*tdh*	exo-2-Methyl-3-methylenebicyclo[2.2.1]heptan-2-ol	−1.000^a^	0.007
*tdh*	2-Pentyl-4-propylthiazole	−1.000^a^	0.008
*trh*	5-(2-Heptadecenyl)-1,3-benzenediol	1.000^a^	0.008
*trh*	Zinc methionine sulfate	−1.000^a^	0.002
*trh*	Zucchini factor B	−1.000^a^	0.002
*trh*	Homoarecoline	−1.000^a^	0.003

^a^Presented correlation is significant at 0.01 level

## Discussion

*V. parahaemolyticus*, a major seafood-derived pathogen which can cause gastrointestinal illness in humans, has attracted more and more interest especially in its virulence in recent years [8, 9]. Two virulence genes expression and metabolites profile were investigated at different incubation temperature. The relationship between them was explored for the first time.

In this study, both *tdh* and *trh* gene expression showed a similar trend, that the expression level reached highest at 25 °C. These results suggested that though 37 °C was regularly used as the culturing temperature in *V. parahaemolyticus* for simulating actual environmental condition in the human gut, 25 °C was more conducive for virulence gene expression in culture medium.

Of thousands of metabolites, 200–300 kinds of them mainly changed, which including low-molecular-weight organic acids, amino acids, alcohols, ketones, esters, sugars and others. These same categories of metabolites were also detected in other microorganism, such as yeast [26] and *Listeria monocytogenes* [27]. Ewald et al. reported that the concentration of intracellular metabolites was determined by the molecular integration of genomic and environmental factors [28]. Meanwhile, the metabolic study on *E. coli* suggested that the concentration of some metabolites changed along with a certain gene mutation in continuous cultures [20]. Thus, the concentration of various metabolites in microorganisms may result from the change of culturing condition and gene expression.

So we hypothesized that there is a correspondence between metabolome and virulence gene expression; and both of them are impacted by culturing temperature.

In this study, based on statistical analysis, 12 and four metabolites showed extremely significant correlation with *tdh* and *trh* gene expression, respectively. The alteration rules of metabolites and virulence gene expression are correlated together by the regulation of culturing temperature. Not much research is available about discussing metabolites and their function in the life cycle of microorganism. Nevertheless we found a few possible connections between several of them or characteristics in metabolic process. 3D, 7D, 11D-Phytanic acid is a branched chain fatty acid, and could be a kind of Fatty-acid metabolic intermediate. It undergoes α-oxidation in the cytosol, where it is converted into pristanic acid by the removal of one carbon [29]. In the third process of α-oxidation, 2-hydroxyphytanoyl-CoA is cleaved by 2-hydroxyphytanoyl-CoA lyase in a TPP-dependent reaction to form pristanal and formyl-CoA. TPP consists of a pyrimidine ring which is connected to a thiazole ring, which is in turn connected to a pyrophosphate functional group. Coincidentally, pyrophosphate changes with 3D, 7D, 11D-Phytanic acid at the same trend in our data suggesting they might be involved in the same metabolic pathway. LysoPE(0:0/16:1(9Z)) is a lysophosphatidylethanolamine (LPE) or a lysophospholipid (LPL). It could transfer to different derivatives by simple enzymatic action. Some LPLs serve important signaling functions [30] in cells and might be a signal molecule for answering the alteration of environmental factors. Homoarecoline, isolated from betel nuts, belongs to the class of organic compounds known as alkaloids and derivatives of arecoline. Arecoline is known to be a partial agonist of muscarinic acetylcholine M1, M2, M3 receptors and M4, [31–33] which is believed to be the primary cause of its parasympathetic effects. Arecoline has also been used medicinally as an antihelmintic [34]. In addition, DG(14:1n5/0:0/20:5n3), DG(20:5n3/0:0/22:6n3), exo-2-Methyl-3-methylenebicyclo[2.2.1]heptan-2-ol and Glucosylceramide (d18:1/25:0), are lipids or lipid-like molecules in *V. parahaemolyticus* ATCC33846; 5-(2-Heptadecenyl)-1,3-benzenediol and zucchini factor B are benzene and substituted derivatives in *V. parahaemolyticus* ATCC17802.

We believe that once increasing experimental evidences are explored in metabolic pathways, these metabolites which have been screened out could be developed as biomarkers or regulators for *V. parahaemolyticus* in future research. New biomarkers would be helpful in monitoring and rapid detection of virulence factors; some metabolites could be used as regulatory factors to control the growth of bacteria; and some potential metabolites might be developed as new drugs to treat diseases caused by *V. parahaemolyticus*.

## Conclusions

This study proved that there is a correlation between the metabolome and virulence gene expression, under different culturing temperatures. This relationship provides a new perspective for better monitoring virulence performance and understanding pathogenesis of this bacteria. Moreover, some certain metabolites could be developed as biomarkers in future research of *Vibrio parahaemolyticus*.

## Methods

### Strains and cultivation

*V. parahaemolyticus* ATCC33846 and ATCC17802 were purchased from the American type culture collection and the stock cultures were maintained at −80 °C in 25 % glycerol solution. The frozen culture was activated in tryptic soy broth (TSB, Beijing Land Bridge Technology Company Ltd., Beijing, PRC) plus 3 % NaCl and incubated at 37 °C with two consecutive transfers after a 10 h incubation. One hundred mL TSB (3 % NaCl) in a 250 mL flask was inoculated with 200 μL inoculum and incubated at 37 °C or 12 h with shaking at 180 r/min, then shifted to 4, 25 and 37 °C statically for 12 h.

### Enumeration of bacteria

Bacteria were counted according to the procedure described in a previous study [35] with some modifications. Briefly, the culture was serially diluted 10-fold in 0.85 % NaCl solution, and then 0.1 mL samples of each dilution were spread onto the thiosulfate citrate bile sucrose agar (TCBS, Beijing Land Bridge Technology Company Ltd., Beijing, PRC) plate. The bacteria counts were enumerated after incubation at 37 °C for 24 h.

### RNA extractions and cDNA synthesis

Cells of the culture (1 mL) were harvested by centrifugation at 12,000 *g* for 5 min, and resuspended in 1 mL Trizol reagent (Invitrogen, Carlsbad, USA) for 15 min and incubated at room temperature for 20 min. Nucleic acids were recovered from the lysate by adding 200 μL solution (phenol: chloroform: isoamyl alcohol = 25: 24: 1) (Sangon, Shanghai, PRC), followed by centrifugation at 12,000 *g* for 15 min at 4 °C. The aqueous layer was then transferred into a clean microcentrifuge tube. Nucleic acids were precipitated by adding equivalent isopropanol (Sangon, Shanghai, PRC) and pelleted by centrifugation at 12,000 *g* for 10 min at 4 °C. The pellet was washed with 75 % cold ethanol (−20 °C) (Sangon, Shanghai, PRC) by centrifuged at 10,000 *g* for 5 min at 4 °C, air dried, and resuspended in 30 μL diethyl pyrocarbonate (DEPC) water (Sangon, Shanghai, PRC). RNA quality was checked on 1 % agarose gel. Then the samples were stored at −80 °C for further analysis.

Reverse transcription (RT) was performed with 200 ng total RNA using the PrimeScript RT reagent Kit with gDNA Eraser (Takara, Dalian, PRC) following the manufacturer’s instructions.

### Quantitative real-time PCR analysis

Relative gene expression was performed by real-time PCR using the ABI 7500 Fast quantitative PCR system (Applied Biosystems, Carlsbad, USA) and FastStart Universal SYBR Green Master (Rox) (Roche, Mannheim, Germany). Primers used in this study were described in Additional file 5: Table S5 and *pvuA* and *pvsA* were used as reference genes [36, 37]. The primers of *tdh* and *trh* gene were referenced the PCR detection method in the FDA bacteriological analytical manual [38]. Amplifications were performed in duplicate. The primers were diluted to 10 μM before use. Each PCR was performed with a 20 μL final volume containing 2 μL cDNA, 1.5 μL (each) primers, 5 μL diH_2_O, 10 μL 1 × SYBR Green PCR Master Mix (Roche). The following thermal cycling conditions were used: a denaturation program (95 °C for 10 min), an amplification program repeated 40 times (95 °C for 15 s and 60 °C for 1 min). Negative controls (deionized water) were included in each run. Melt curve analysis was performed on the PCR products at the end of each run to ensure that a single product was amplified. Relative quantification was measured using the 2^-ΔΔCt^ method (the amount of target, normalized to an endogenous control and relative to a calibrator, where ΔΔCt = (Ct _target_ − Ct _reference_) _sample_ − (Ct _target_ − Ct _reference_) _calibrator_) [39]. The C_t_ is the number of cycles needed for the fluorescence signal to reach a specific threshold level of detection and is negatively correlated with the amount of template nucleic acid in the reaction. All values are the normalized means ± standard deviations (SD) of the results for two runs, each with two replicate samples.

### Sampling for intracellular metabolites

Approximately 5 × 10^8^ CFU of bacteria were injected into a tube containing 15 mL pre-cooled solution of 75 % methanol (v/v) with 70 mM 4-(2-hydroxyethyl)-1-piperazineethanesulfonic acid (HEPES) (−80 °C). The contents of tube were quickly mixed by vortexing and then the tube was stored in the ice for 5 min.

### Metabolite extraction

Extraction of intracellular metabolites was performed using the cold ethanol method of Buchholz et al. [40] with some modifications. Briefly, the cells were centrifuged at 4,650 *g* for 10 min with a pre-cooled rotor of 4 °C. The cell pellet was resuspended in 1 mL of cold 75 % methanol (−20 °C). After rapid mixing, the mixture was frozen at −80 °C for 5 min and thawed at 65 °C for 10 min, which was performed with two consecutive repetitions. After the freeze-thaw cycle, proteins and cell fragments were removed by centrifugation at 12,000 *g* for 2 min at 4 °C. The supernatant was stored at −80 °C until further analysis.

### Chromatography

Chromatographic separations were performed on an ACQUITY™ UPLC System (Waters Corporation, Milford, MA). A BEH C18 reversed-phase column (100 × 2.1 mm, 1.7 μm, Waters, MA, USA) and a BEH C18 guard column (5 × 2.1 mm, 1.7 μm) were used. The column was maintained at 37 °C with a flow rate of 0.4 mL/min. Mobile phase A was 0.1 % formic acid (Sinopharm, Shanghai, PRC), while mobile phase B was acetonitrile (Sinopharm, Shanghai, PRC) modified by addition of 0.1 % formic acid. Each sample was run twice: once in positive ionisation mode and once in negative ionisation mode. In positive mode, the gradient was t = 0 min, 99 % B; t = 2 min, 70 % B; t = 4 min, 25 % B; t = 7 min, 25 % B; t = 9 min, 0 % B; t = 11.5 min, 0 % B; t = 12 min, 99 % B; t = 13.5 min, 99 % B. In negative mode, the gradient was t = 0 min, 99 % B; t = 2 min, 70 % B; t = 4 min, 25 % B; t = 5 min, 25 % B; t = 7 min, 10 % B; t = 8 min, 0 % B; t = 10 min, 0 % B; t = 10.4 min, 99 % B; t = 12.2 min, 99 % B.

### Mass spectrometry

MS spectrometry was carried out on a Water Q-TOF Primer system (Waters Corporation, Milford, MA) with electrospray source ionization (ESI) operation in both positive and negative ion ionisation modes. Nitrogen was used as the drying gas. For both positive and negative ionisation modes, the capillary and conevoltage were set at 3 kV and 55 V, respectively. The desolvation gas was set to 650 L/h at a temperature of 350 °C, and the cone gas was set to 50 L/h and the source temperature was set to 100 °C. The data acquisition rate was set to 0.28 s, with a 0.02 s interscan delay. Data was acquired with a scan range from 50 to 1000 Da.

### Data analysis

The RT-qPCR data were analyzed using the ABI 7500 fast system. The quantity results based on RT-qPCR for *tdh* and *trh* genes at 25 °C were used as datum for relative quantity data, which were respectively set as to 1. The RT-qPCR value of *tdh* or *trh* genes for other sample was converted to relative quantity data in comparison with the value from datum. A one-way ANOVA was performed by Microsoft office Excel 2007 (Microsoft, Redmond, USA) to determine significant differences at α = 0.05.

UPLC-MS spectra data were first processed by Markerlynx Applications Manager Version 4.1 (Waters, Manchester, UK), including the detection and retention time (R.T.) alignment of peaks in each chromatogram. Metabolites were identified by mass-to-charge ratios in the human metabolome database (HMDB). The processed data were then introduced to SIMCA-P 11.5 (Umetrics, Umea, Sweden). Multivariate statistical analysis method of principal component analysis (PCA) was performed to determine the trend of data which transforms the correlated variables dataset into a smaller number of independent variables, i.e., the principle components [41].

Pearson’s correlation analysis was performed using the SPSS 17.0 (SPSS Inc., Chicago, USA). The correlation analysis was performed between the virulence genes expression and metabolome.

## Declarations

### Ethic approval and consent to participate

Not applicable.

### Consent for publication

Not applicable.

### Availability of data and materials

The data sets supporting the results of this article are included within the article and its additional file.

## Additional files

Additional file 1: Table S1. Relative concentration of metabolites identified in *Vibrio parahaemolyticus* ATCC33846. (XLSX 123 kb). Additional file 2: Table S2. Relative concentration of metabolites identified in *Vibrio parahaemolyticus* ATCC17802. (XLSX 113 kb). Additional file 3: Table S3. Chemical taxonomy of significantly changed metabolitesof *Vibrio parahaemolyticus* ATCC33846. (DOCX 20 kb). Additional file 4: Table S4. Chemical taxonomy of significantly changed metabolites of *Vibrio parahaemolyticus* ATCC17802. (DOCX 17 kb). Additional file 5: Table S5. Primers sequences used in RT-qPCR. (DOCX 14 kb).

## Abbreviations

*V. parahaemolyticus*: *vibrio parahaemolyticus*
ATCC: American type culture collection
PCA: principal components analysis
UPLC & Q-TOF MS: ultra-performance liquid chromatography & quadrupole time-of-flight mass spectrometry
TDH: thermostable direct hemolysin
*tdh*: thermostable direct hemolysin gene
TRH: TDH-related hemoysin
*trh*: TDH-related hemoysin gene
T3SS: type III secretion systems
T6SS: type VI secretion systems
PC1: first principal component
PC2: second principal component
HMDB: human metabolome database
CoA: coenzyme A
TPP: triphenyl phosphate
LPE: lysophosphatidylethanolamine
LPL: lysophospholipid
DG: diacylglycerol
TSB: tryptic soy broth
TCBS: thiosulfate citrate bile sucrose agar
DEPC: diethyl pyrocarbonate
HEPES: 4-(2-hydroxyethyl)-1-piperazineethanesulfonic acid
