# Supplementary material for: Metabolome response to temperature-induced virulence gene expression in two genotypes of pathogenic Vibrio parahaemolyticus
Source: BMC Microbiol. 2016 Apr 26;16:75. doi: 10.1186/s12866-016-0688-5 (PMC4845332; doi:10.1186/s12866-016-0688-5)
Supplement: Additional file 3: Table S3. — Chemical taxonomy of significantly changed metabolitesof Vibrio parahaemolyticus ATCC33846. (DOCX 20 kb). [file 12866_2016_688_MOESM3_ESM.docx]

**Table S3** Chemical taxonomy of significantly changed metabolites of *Vibrio parahaemolyticus* ATCC33846.

| Metabolites | Super Class | Class |
| --- | --- | --- |
| APC | Alkaloids and derivatives | Camptothecins |
| Cabergoline | Alkaloids and derivatives | Ergoline and derivatives |
| Dihydroergotoxine | Alkaloids and derivatives | Ergoline and derivatives |
| 2-Hydroxy-3-(3,4-dihydroxyphenyl)propanamide | Benzenoids | Benzene and substituted derivatives |
| 2-phthalimidoglutaric acid | Benzenoids | Benzene and substituted derivatives |
| 3-(10-Heptadecenyl)phenol | Benzenoids | Benzene and substituted derivatives |
| 5-(2-Heptadecenyl)-1,3-benzenediol | Benzenoids | Benzene and substituted derivatives |
| Bepridil | Benzenoids | Benzene and substituted derivatives |
| Cinnamyl benzoate | Benzenoids | Benzene and substituted derivatives |
| Darifenacin | Benzenoids | Benzene and substituted derivatives |
| Isopropamide | Benzenoids | Benzene and substituted derivatives |
| Methyldopa | Benzenoids | Benzene and substituted derivatives |
| Niclosamide | Benzenoids | Benzene and substituted derivatives |
| Tolterodine | Benzenoids | Benzene and substituted derivatives |
| Trabectedin metabolite M16e | Benzenoids | Benzene and substituted derivatives |
| (8R,8'R)-Secoisolariciresinol 9,9'-bis-[4-carboxy-3-hydroxy-3-methylbutanoyl-(->6)-glucoside] | Lignans, neolignans and related compounds | Lignan glycosides |
| [6]-Gingerdiol 3,5-diacetate | Lipids and lipid-like molecules | Fatty Acyls |
| 2,3-Methylenesuccinic acid | Lipids and lipid-like molecules | Fatty Acyls |
| 2,4,12-Octadecatrienoic acid isobutylamide | Lipids and lipid-like molecules | Fatty Acyls |
| 2-Acetolactate | Lipids and lipid-like molecules | Fatty Acyls |
| 3, 5-Tetradecadiencarnitine | Lipids and lipid-like molecules | Fatty Acyls |
| 6S,9R-Dihydroxy-4,7E-megastigmadien-3-one 9-[apiosyl-(1->6)-glucoside] | Lipids and lipid-like molecules | Fatty Acyls |
| 9,12-Hexadecadienoylcarnitine | Lipids and lipid-like molecules | Fatty Acyls |
| Arachidonic acid | Lipids and lipid-like molecules | Fatty Acyls |
| Arachidonoyl Serinol | Lipids and lipid-like molecules | Fatty Acyls |
| Benzoyl-CoA | Lipids and lipid-like molecules | Fatty Acyls |
| cis-5-Tetradecenoylcarnitine | Lipids and lipid-like molecules | Fatty Acyls |
| Hexyl octanoate | Lipids and lipid-like molecules | Fatty Acyls |
| Tetradecanoyl-CoA | Lipids and lipid-like molecules | Fatty Acyls |
| Triacetin | Lipids and lipid-like molecules | Glycerolipids |
| 6-(N-Acetyl-alpha-D-glucosaminyl)-1-phosphatidyl-1D-myo-inositol | Lipids and lipid-like molecules | Glycerophospholipids |
| Glycerol 3-phosphate | Lipids and lipid-like molecules | Glycerophospholipids |
| LPA(0:0/18:0) | Lipids and lipid-like molecules | Glycerophospholipids |
| LysoPC(16:0) | Lipids and lipid-like molecules | Glycerophospholipids |
| PA(20:4(5Z,8Z,11Z,14Z)e/2:0) | Lipids and lipid-like molecules | Glycerophospholipids |
| PC(o-18:1(9Z)/22:0) | Lipids and lipid-like molecules | Glycerophospholipids |
| PE(15:0/18:3(9Z,12Z,15Z)) | Lipids and lipid-like molecules | Glycerophospholipids |
| PE(16:1(9Z)/18:0) | Lipids and lipid-like molecules | Glycerophospholipids |
| PE(18:2(9Z,12Z)/22:2(13Z,16Z)) | Lipids and lipid-like molecules | Glycerophospholipids |
| PE(20:4(8Z,11Z,14Z,17Z)/16:1(9Z)) | Lipids and lipid-like molecules | Glycerophospholipids |
| PE(22:0/24:0) | Lipids and lipid-like molecules | Glycerophospholipids |
| PIP2(16:0/18:1(11Z)) | Lipids and lipid-like molecules | Glycerophospholipids |
| PS(18:2(9Z,12Z)/18:3(9Z,12Z,15Z)) | Lipids and lipid-like molecules | Glycerophospholipids |
| PS(18:3(9Z,12Z,15Z)/22:6(4Z,7Z,10Z,13Z,16Z,19Z)) | Lipids and lipid-like molecules | Glycerophospholipids |
| sn-glycero-3-Phosphoethanolamine | Lipids and lipid-like molecules | Glycerophospholipids |
| Linolenelaidic acid | Lipids and lipid-like molecules | Lineolic acids and derivatives |
| Methyl linoleate | Lipids and lipid-like molecules | Lineolic acids and derivatives |
| MG(18:2(9Z,12Z)/0:0/0:0) | Lipids and lipid-like molecules | Lineolic acids and derivatives |
| MG(18:3(9Z,12Z,15Z)/0:0/0:0) | Lipids and lipid-like molecules | Lineolic acids and derivatives |
| MG(18:4(6Z,9Z,12Z,15Z)/0:0/0:0) | Lipids and lipid-like molecules | Lineolic acids and derivatives |
| MG(20:1(11Z)/0:0/0:0) | Lipids and lipid-like molecules | Lineolic acids and derivatives |
| 10'-Apo-beta-caroten-10'-al | Lipids and lipid-like molecules | Prenol lipids |
| 13'-Hydroxy-alpha-tocotrienol | Lipids and lipid-like molecules | Prenol lipids |
| Cinncassiol C1 19-glucoside | Lipids and lipid-like molecules | Prenol lipids |
| Crocin | Lipids and lipid-like molecules | Prenol lipids |
| Eremopetasitenin D2 | Lipids and lipid-like molecules | Prenol lipids |
| Ganoderic acid N | Lipids and lipid-like molecules | Prenol lipids |
| Glabrolide | Lipids and lipid-like molecules | Prenol lipids |
| Pitheduloside B | Lipids and lipid-like molecules | Prenol lipids |
| Pristanoylglycine | Lipids and lipid-like molecules | Prenol lipids |
| Trimethyltridecanoic acid | Lipids and lipid-like molecules | Prenol lipids |
| Eucaglobulin | Lipids and lipid-like molecules | Saccharolipids |
| Dihydroceramide | Lipids and lipid-like molecules | Sphingolipids |
| SM(d18:1/26:0) | Lipids and lipid-like molecules | Sphingolipids |
| (22Alpha)-hydroxy-campest-4-en-3-one | Lipids and lipid-like molecules | Steroids and steroid derivatives |
| 24-Methylenepollinastanone | Lipids and lipid-like molecules | Steroids and steroid derivatives |
| 2-Hydroxydesogestrel | Lipids and lipid-like molecules | Steroids and steroid derivatives |
| 3beta-Hydroxypregn-5-en-20-one sulfate | Lipids and lipid-like molecules | Steroids and steroid derivatives |
| 3-Epidemissidine | Lipids and lipid-like molecules | Steroids and steroid derivatives |
| 5alpha-Tomatidan-3-one | Lipids and lipid-like molecules | Steroids and steroid derivatives |
| 6-beta-Hydroxy-mometasone furoate | Lipids and lipid-like molecules | Steroids and steroid derivatives |
| 7-Ketodeoxycholic acid | Lipids and lipid-like molecules | Steroids and steroid derivatives |
| Alliospiroside D | Lipids and lipid-like molecules | Steroids and steroid derivatives |
| Epiandrosterone | Lipids and lipid-like molecules | Steroids and steroid derivatives |
| Neoconvallatoxoloside | Lipids and lipid-like molecules | Steroids and steroid derivatives |
| Norethindrone | Lipids and lipid-like molecules | Steroids and steroid derivatives |
| Physalolactone | Lipids and lipid-like molecules | Steroids and steroid derivatives |
| Portensterol | Lipids and lipid-like molecules | Steroids and steroid derivatives |
| Varanic acid | Lipids and lipid-like molecules | Steroids and steroid derivatives |
| Guanosine monophosphate | Nucleosides, nucleotides, and analogues | Purine nucleotides |
| 2,4,8-Eicosatrienoic acid isobutylamide | Organic acids and derivatives | Carboximidic acids and derivatives |
| Neoherculin | Organic acids and derivatives | Carboximidic acids and derivatives |
| Asparaginyl-Proline | Organic acids and derivatives | Carboxylic acids and derivatives |
| Iminoaspartic acid | Organic acids and derivatives | Carboxylic acids and derivatives |
| Morphiceptin | Organic acids and derivatives | Carboxylic acids and derivatives |
| N-Acetyl-L-glutamate 5-semialdehyde | Organic acids and derivatives | Carboxylic acids and derivatives |
| N-Decanoylglycine | Organic acids and derivatives | Carboxylic acids and derivatives |
| Neocasomorphin | Organic acids and derivatives | Carboxylic acids and derivatives |
| N-Lauroylglycine | Organic acids and derivatives | Carboxylic acids and derivatives |
| Oleoyl glycine | Organic acids and derivatives | Carboxylic acids and derivatives |
| Rigin | Organic acids and derivatives | Carboxylic acids and derivatives |
| Selenocystine | Organic acids and derivatives | Carboxylic acids and derivatives |
| Stepronin | Organic acids and derivatives | Carboxylic acids and derivatives |
| Sucrose acetate isobutyrate | Organic acids and derivatives | Carboxylic acids and derivatives |
| Hydroxypyruvic acid | Organic acids and derivatives | Hydroxy acids and derivatives |
| 2-Keto-6-acetamidocaproate | Organic acids and derivatives | Keto acids and derivatives |
| D-Arginine | Organic acids and derivatives | Organic acids and derivatives |
| 1,2-Dichloroethane | Organohalogen compounds | Organochlorides |
| Azaspiracid 5 | Organoheterocyclic compounds | Azaspirodecane derivatives |
| Cyclovariegatin | Organoheterocyclic compounds | Benzofurans |
| Nefazodone | Organoheterocyclic compounds | Diazinanes |
| Asparagusic acid syn-S-oxide | Organoheterocyclic compounds | Dithiolanes |
| 4-[(2-Furanylmethyl)thio]-2-pentanone | Organoheterocyclic compounds | Heteroaromatic compounds |
| Cefotetan | Organoheterocyclic compounds | Lactams |
| O-Demethylfonsecin | Organoheterocyclic compounds | Naphthopyrans |
| PC-M6 | Organoheterocyclic compounds | Naphthopyrans |
| 2,4,12-Octadecatrienoic acid piperidide | Organoheterocyclic compounds | Piperidines |
| 5-Methyltetrahydropteroylpentaglutamate | Organoheterocyclic compounds | Pteridines and derivatives |
| 4-Pyridoxic acid | Organoheterocyclic compounds | Pyridines and derivatives |
| Nicardipine | Organoheterocyclic compounds | Pyridines and derivatives |
| Pyridoxamine | Organoheterocyclic compounds | Pyridines and derivatives |
| 1-Pyrroline-4-hydroxy-2-carboxylate | Organoheterocyclic compounds | Pyrrolines |
| Citbismine C | Organoheterocyclic compounds | Quinolines and derivatives |
| Heme A | Organoheterocyclic compounds | Tetrapyrroles and derivatives |
| Porphobilinogen | Organonitrogen compounds | Amines |
| (3x,5x,10x)-9,10-Didehydroisohumbertiol O-[rhamnosyl-(1->4)-rhamnosyl-(1->2)-[rhamnosyl-(1->6)]-glucoside] | Organooxygen compounds | Carbohydrates and carbohydrate conjugates |
| 3-Sialyl-N-acetyllactosamine | Organooxygen compounds | Carbohydrates and carbohydrate conjugates |
| 6-Methyl 2-galloylgalactarate | Organooxygen compounds | Carbohydrates and carbohydrate conjugates |
| Chitin | Organooxygen compounds | Carbohydrates and carbohydrate conjugates |
| LS tetrasaccharide d | Organooxygen compounds | Carbohydrates and carbohydrate conjugates |
| Neomycin | Organooxygen compounds | Carbohydrates and carbohydrate conjugates |
| N-Sulfo-D-glucosamine | Organooxygen compounds | Carbohydrates and carbohydrate conjugates |
| 1-Mercapto-5-propanone | Organooxygen compounds | Carbonyl compounds |
| 2,2-Dihydroperoxypropane | Organooxygen compounds | Organic hydroperoxides |
| Isoflurophate | Organophosphorus compounds | Organic phosphoric acids and derivatives |
| N-Methylethanolaminium phosphate | Organophosphorus compounds | Organic phosphoric acids and derivatives |
| Sphingosine 1-phosphate (d16:1-P) | Organophosphorus compounds | Organic phosphoric acids and derivatives |
| Methanedithiol | Organosulfur compounds | Thiols |
| Caffeoylferuloylspermidine | Phenylpropanoids and polyketides | Cinnamic acids and derivatives |
| Furcelleran | Phenylpropanoids and polyketides | Cinnamic acids and derivatives |
| 9-Hydroxy-4-methoxypsoralen 9-glucoside | Phenylpropanoids and polyketides | Coumarins and derivatives |
| Celereoin | Phenylpropanoids and polyketides | Coumarins and derivatives |
| 5-Methoxy-7-(4-hydroxyphenyl)-1-phenyl-3-heptanone | Phenylpropanoids and polyketides | Diarylheptanoids |
| (-)-Catechin 3-O-gallate | Phenylpropanoids and polyketides | Flavonoids |
| (-)-Epigallocatechin 3-gallate 7-glucoside 4"-glucuronide | Phenylpropanoids and polyketides | Flavonoids |
| Delphinidin 3-lathyroside 5-glucoside | Phenylpropanoids and polyketides | Flavonoids |
| Dihydromorelloflavone | Phenylpropanoids and polyketides | Flavonoids |
| Flavoxate | Phenylpropanoids and polyketides | Flavonoids |
| Spinacetin 3-(2''-apiosylgentiobioside) | Phenylpropanoids and polyketides | Flavonoids |
| 7-Hydroxy-2',5,6-trimethoxy-4',5'-methylenedioxyisoflavone 7-(2-p-coumaroylglucoside) | Phenylpropanoids and polyketides | Isoflavonoids |
| Luteone 7-glucoside | Phenylpropanoids and polyketides | soflavonoids |
| 3-O-beta-D-Galactopyranosylproanthocyanidin A5' | Phenylpropanoids and polyketides | Tannins |
| Prodelphinidin B | Phenylpropanoids and polyketides | Tannins |
| (1alpha,3beta,20S,22R,24S,25S)-Pubescenin | ­- | - |
| (3beta,22E,24R)-Ergosta-4,6,8(14),22-tetraen-3-ol | - | - |
| 11-peroxy-5Z,8Z,12E,14Z-eicosatetraenoate | - | - |
| Abscisic alcohol 11-glucoside | - | - |
| alpha-(o-carboxybenzamido)glutarimide | - | - |
| alpha-Hydroxytamoxifen N-oxide | - | - |
| Artonol C | - | - |
| Avocadyne | - | - |
| Avocadyne 1-acetate | - | - |
| Benzoin | - | - |
| beta-Citraurol | - | - |
| Bipindogulomethyloside | - | - |
| Chatenaytrienin 1 | - | - |
| Chinenoside VI | - | - |
| Citrusin F | - | - |
| D-Erythroascorbic acid 1'-a-D-xylopyranoside | - | - |
| DG(15:0/0:0/16:1n7) | - | - |
| DG(15:0/0:0/22:4n6) | - | - |
| DG(18:0/0:0/18:2n6) | - | - |
| Dihydromaleimide beta-D-glucoside | - | - |
| epsilon-Tocopherol | - | - |
| Ferrous gluconate | - | - |
| Ganodosterone | - | - |
| Goshonoside F2 | - | - |
| Isopentyl beta-D-glucoside | - | - |
| Kanokoside A | - | - |
| Mucronine D | - | - |
| Pangamic acid | - | - |
| Pectenotoxin 1 | - | - |
| Piceatannol 4'-galloylglucoside | - | - |
| Quercetin 3-(2-galloylglucoside) | - | - |
| Rebaudioside A | - | - |
| Riboflavine 2',3',4',5'-tetrabutanoate | - | - |
| Stevioside | - | - |
| Tartrazine calcium lake | - | - |
| TG(15:0/14:0/16:1(9Z)) | - | - |

-: unknown
