# Supplementary material for: Metabolome response to temperature-induced virulence gene expression in two genotypes of pathogenic Vibrio parahaemolyticus
Source: BMC Microbiol. 2016 Apr 26;16:75. doi: 10.1186/s12866-016-0688-5 (PMC4845332; doi:10.1186/s12866-016-0688-5)
Supplement: Additional file 4: Table S4. — Chemical taxonomy of significantly changed metabolites of Vibrio parahaemolyticus ATCC17802. (DOCX 17 kb). [file 12866_2016_688_MOESM4_ESM.docx]

**Table S4** Chemical taxonomy of significantly changed metabolites of *Vibrio parahaemolyticus* ATCC17802.

| Metabolites | Super class | Class |
| --- | --- | --- |
| 5-(14-Nonadecenyl)-1,3-benzenediol | Benzenoids | Benzene and substituted derivatives |
| 5-(2-Heptadecenyl)-1,3-benzenediol | Benzenoids | Benzene and substituted derivatives |
| C.I. Acid Green 3 | Benzenoids | Benzene and substituted derivatives |
| Cinnamyl benzoate | Benzenoids | Benzene and substituted derivatives |
| Magnesium salicylate | Benzenoids | Benzene and substituted derivatives |
| Tioconazole | Benzenoids | Benzene and substituted derivatives |
| Trabectedin metabolite M16e | Benzenoids | Benzene and substituted derivatives |
| Argenteane | Lignans, neolignans and related compounds | Dibenzylbutane lignans |
| (8R,8'R)-Secoisolariciresinol 9,9'-bis-[4-carboxy-3-hydroxy-3-methylbutanoyl-(->6)-glucoside] | Lignans, neolignans and related compounds | Lignan glycosides |
| 2-Acetolactate | Lipids and lipid-like molecules | Fatty Acyls |
| Leukotriene D4 | Lipids and lipid-like molecules | Fatty Acyls |
| Nonanoyl-CoA | Lipids and lipid-like molecules | Fatty Acyls |
| Tetradecanoyl-CoA | Lipids and lipid-like molecules | Fatty Acyls |
| DG(22:5(4Z,7Z,10Z,13Z,16Z)/22:5(7Z,10Z,13Z,16Z,19Z)/0:0) | Lipids and lipid-like molecules | Glycerolipids |
| Triacetin | Lipids and lipid-like molecules | Glycerolipids |
| LysoPE(18:1(9Z)/0:0) | Lipids and lipid-like molecules | Glycerophospholipids |
| PC(18:4(6Z,9Z,12Z,15Z)/18:3(9Z,12Z,15Z)) | Lipids and lipid-like molecules | Glycerophospholipids |
| PC(22:5(4Z,7Z,10Z,13Z,16Z)/22:5(7Z,10Z,13Z,16Z,19Z)) | Lipids and lipid-like molecules | Glycerophospholipids |
| PE(18:0/20:5(5Z,8Z,11Z,14Z,17Z)) | Lipids and lipid-like molecules | Glycerophospholipids |
| PGP(18:1(9Z)/18:0) | Lipids and lipid-like molecules | Glycerophospholipids |
| PIP2(16:0/18:1(11Z)) | Lipids and lipid-like molecules | Glycerophospholipids |
| 20-Hydroxy-3,7,11,15,23-pentaoxolanost-8-en-26-oic acid | Lipids and lipid-like molecules | Prenol lipids |
| Eremopetasitenin D2 | Lipids and lipid-like molecules | Prenol lipids |
| Ipomeatetrahydrofuran | Lipids and lipid-like molecules | Prenol lipids |
| Momordin I | Lipids and lipid-like molecules | Prenol lipids |
| Pristanoylglycine | Lipids and lipid-like molecules | Prenol lipids |
| Trimethyltridecanoic acid | Lipids and lipid-like molecules | Prenol lipids |
| Glucosylsphingosine | Lipids and lipid-like molecules | Sphingolipids |
| Sphinganine | Lipids and lipid-like molecules | Sphingolipids |
| 11-beta-Hydroxyandrosterone-3-glucuronide | Lipids and lipid-like molecules | Steroids and steroid derivatives |
| 25-Acetyl-6,7-didehydrofevicordin F 3-[glucosyl-(1->6)-glucoside] | Lipids and lipid-like molecules | Steroids and steroid derivatives |
| 25-Acetyl-6,7-didehydrofevicordin F 3-glucoside | Lipids and lipid-like molecules | Steroids and steroid derivatives |
| 3-Epidemissidine | Lipids and lipid-like molecules | Steroids and steroid derivatives |
| 5alpha-Tomatidan-3-one | Lipids and lipid-like molecules | Steroids and steroid derivatives |
| Crustecdysone | Lipids and lipid-like molecules | Steroids and steroid derivatives |
| Portensterol | Lipids and lipid-like molecules | Steroids and steroid derivatives |
| P1,P4-Bis(5'-xanthosyl) tetraphosphate | Nucleosides, nucleotides, and analogues | (5'->5')-dinucleotides |
| ADP-Ribosyl-L-arginine | Nucleosides, nucleotides, and analogues | Purine nucleotides |
| Uridine | Nucleosides, nucleotides, and analogues | Pyrimidine nucleosides |
| Asparaginyl-Proline | Organic acids and derivatives | Carboxylic acids and derivatives |
| D-Arginine | Organic acids and derivatives | Carboxylic acids and derivatives |
| Glabrin D | Organic acids and derivatives | Carboxylic acids and derivatives |
| Hydroxy Ritonavir | Organic acids and derivatives | Carboxylic acids and derivatives |
| Iminoaspartic acid | Organic acids and derivatives | Carboxylic acids and derivatives |
| L-Alloisoleucine | Organic acids and derivatives | Carboxylic acids and derivatives |
| Phomopsin B | Organic acids and derivatives | Carboxylic acids and derivatives |
| Cinnavalininate | Organoheterocyclic compounds | Benzoxazines |
| 3-Acetylpyridine | Organoheterocyclic compounds | Diazines |
| Ascladiol | Organoheterocyclic compounds | Dihydrofurans |
| Indoleacrylic acid | Organoheterocyclic compounds | Indoles and derivatives |
| Tubocurarine | Organoheterocyclic compounds | Isoquinolines and derivatives |
| 27-O-demethylrifabutin | Organoheterocyclic compounds | Naphthofurans |
| O-Demethylfonsecin | Organoheterocyclic compounds | Naphthopyrans |
| Pipereicosalidine | Organoheterocyclic compounds | Piperidines |
| Piperidine | Organoheterocyclic compounds | Piperidines |
| 4-Pyridoxic acid | Organoheterocyclic compounds | Pyridines and derivatives |
| Nicardipine | Organoheterocyclic compounds | Pyridines and derivatives |
| Pyridoxamine | Organoheterocyclic compounds | Pyridines and derivatives |
| 1-Pyrroline-4-hydroxy-2-carboxylate | Organoheterocyclic compounds | Pyrrolines |
| Drotaverine | Organoheterocyclic compounds | Tetrahydroisoquinolines |
| N-Gluconyl ethanolamine | Organonitrogen compounds | Amines |
| Porphobilinogen | Organonitrogen compounds | Amines |
| Myo-inositol hexakisphosphate | Organooxygen compounds | Alcohols and polyols |
| Cassiaside B2 | Organooxygen compounds | Carbohydrates and carbohydrate conjugates |
| Chitin | Organooxygen compounds | Carbohydrates and carbohydrate conjugates |
| LS tetrasaccharide d | Organooxygen compounds | Carbohydrates and carbohydrate conjugates |
| N,N'-Diacetylchitobiosyldiphosphodolichol | Organooxygen compounds | Carbohydrates and carbohydrate conjugates |
| Paromomycin | Organooxygen compounds | Carbohydrates and carbohydrate conjugates |
| Dichlorvos | Organophosphorus compounds | Organic phosphoric acids and derivatives |
| Isoflurophate | Organophosphorus compounds | Organic phosphoric acids and derivatives |
| Di-2-propenyl pentasulfide | Organosulfur compounds | Sulfenyl compounds |
| Fenbendazole | Organosulfur compounds | Thioethers |
| cis-Coutaric acid | Phenylpropanoids and polyketides | Cinnamic acids and derivatives |
| Furcelleran | Phenylpropanoids and polyketides | Cinnamic acids and derivatives |
| Cyanidin 3-(malonylsophoroside) 5-glucoside | Phenylpropanoids and polyketides | Flavonoids |
| Delphinidin 3-lathyroside 5-glucoside | Phenylpropanoids and polyketides | Flavonoids |
| Dihydromorelloflavone | Phenylpropanoids and polyketides | Flavonoids |
| Eriodictyol 7-(6-galloylglucoside) | Phenylpropanoids and polyketides | Flavonoids |
| Kaempferol 7-(6''-galloylglucoside) | Phenylpropanoids and polyketides | Flavonoids |
| Spinacetin 3-(2''-apiosylgentiobioside) | Phenylpropanoids and polyketides | Flavonoids |
| (3beta,22E,24R)-Ergosta-4,6,8(14),22-tetraen-3-ol | - | - |
| 3-Hydroxyphenyllactate | - | - |
| Acevaltrate | - | - |
| alpha-Fluoro-beta-alanine | - | - |
| Benzoin | - | - |
| beta-Citraurol | - | - |
| Chinenoside VI | - | - |
| Cytochalasin Opho | - | - |
| D-Erythroascorbic acid 1'-a-D-xylopyranoside | - | - |
| Desglucocheirotoxol | - | - |
| ent-6R,16bOH,17-Trihydroxy-7-oxo-6,7-seco-19,6-kauranolide 6-O-glucoside | - | - |
| erythro-6,8-Tricosanediol | - | - |
| Ethyl 5-oxotetrahydro-2-furancarboxylate | - | - |
| Gibberellin A38 glucosyl ester | - | - |
| Hebevinoside XIV | - | - |
| Isopentyl beta-D-glucoside | - | - |
| Jurubine | - | - |
| Kanokoside A | - | - |
| Quercetin 3-(2-galloylglucoside) | - | - |
| Stevioside | - | - |
| Ustiloxin A | - | - |

-: unknown
