# Supplementary material for: Metabolome response to temperature-induced virulence gene expression in two genotypes of pathogenic Vibrio parahaemolyticus
Source: BMC Microbiol. 2016 Apr 26;16:75. doi: 10.1186/s12866-016-0688-5 (PMC4845332; doi:10.1186/s12866-016-0688-5)
Supplement: Additional file 5: Table S5. — Primers sequences used in RT-qPCR. (DOCX 14 kb). [file 12866_2016_688_MOESM5_ESM.docx]

**Table S5** Primers used in RT-qPCR for *V. parahaemolyticus*.

| Gene | Primer sequence (5′to 3′) |
| --- | --- |
| *pvuA* | F1–*pvuA*: CAAACTCACTCAGACTCCA |
|  | R1–*pvuA*: CGAACCGAT TCAACACG |
| *pvsA* | F2–*pvsA*: CTCCTTCATCCAACACGAT |
|  | R2–*pvsA*: GGGCGAGATAATCCTTGT |
| *trh* | F–*trh*: TTGGCTTCGATATTTTCAGTATCT |
|  | R–*trh*: CATAACAAACATATGCCCATTTCCG |
| *tdh* | F–*tdh*: GTAAAGGTCTCTGACTTTTGGAC |
|  | R–*tdh*: TGGAATAGAACCTTCATCTTCACC |
